# Supplementary material for: Transcriptomic Insights into Mechanisms of Early Seed Maturation in the Garden Pea (Pisum sativum L.)
Source: Cells. 2020 Mar 23;9(3):779. doi: 10.3390/cells9030779 (PMC7140803; doi:10.3390/cells9030779)
Supplement: Supplementary file 1 [file cells-09-00779-s001.zip › Supplementary materials/FileS3.zip!/FileS2/Hormones - Results/Summary Sheets - Groups/Group Assignment.pdf]

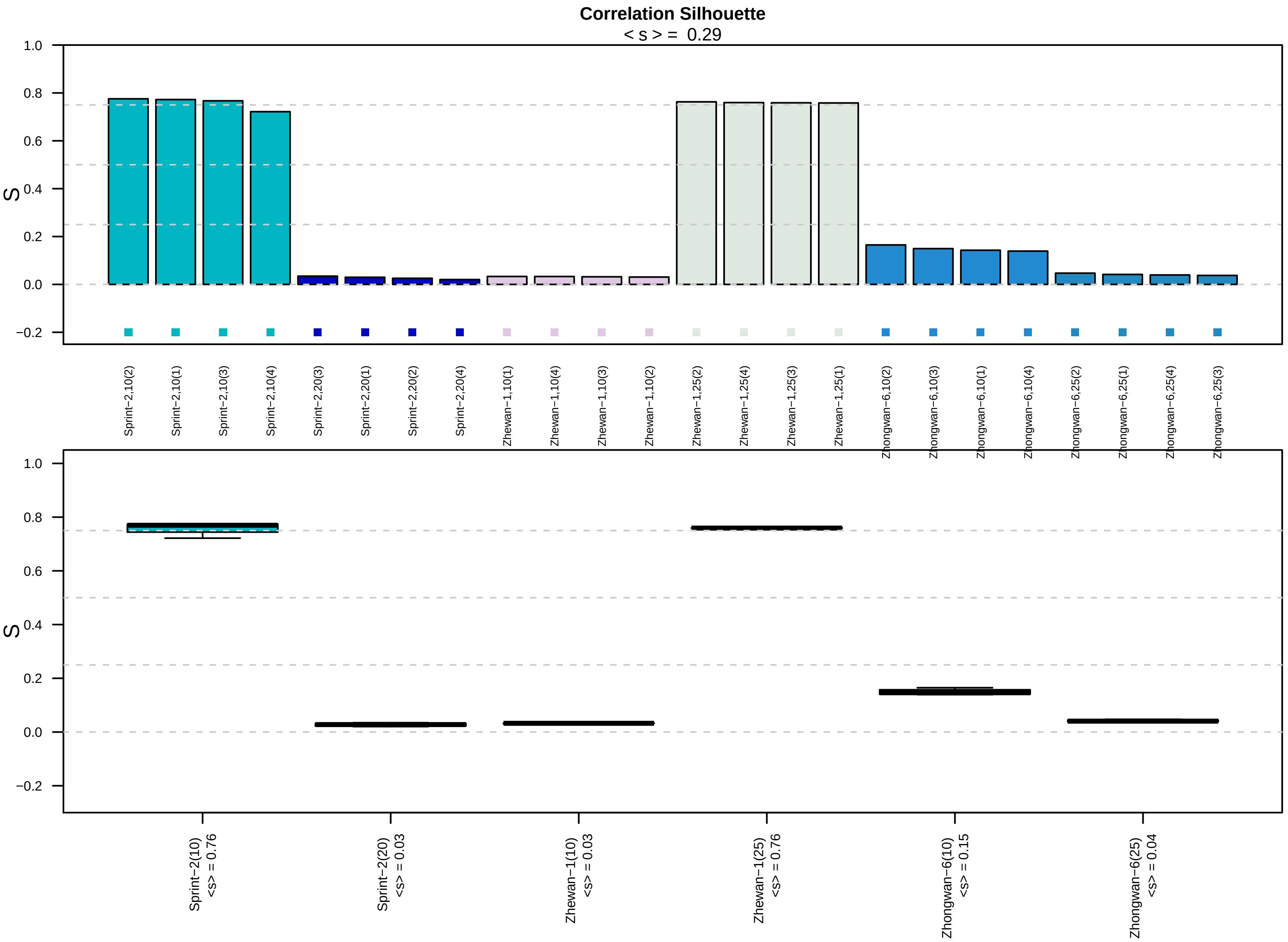

Correlation Silhouette
< s > = 0.29
S
1.0
0.8
0.6
0.4
0.2
0.0
−0.2
Sprint−2,10(2)
Sprint−2,10(1)
Sprint−2,10(3)
Sprint−2,10(4)
Sprint−2,20(3)
Sprint−2,20(1)
Sprint−2,20(2)
Sprint−2,20(4)
Zhewan−1,10(1)
Zhewan−1,10(4)
Zhewan−1,10(3)
Zhewan−1,10(2)
Zhewan−1,25(2)
Zhewan−1,25(4)
Zhewan−1,25(3)
Zhewan−1,25(1)
Zhongwan−6,10(2)
Zhongwan−6,10(3)
Zhongwan−6,10(1)
Zhongwan−6,10(4)
Zhongwan−6,25(2)
Zhongwan−6,25(1)
Zhongwan−6,25(4)
Zhongwan−6,25(3)
Sprint−2(10) <s> = 0.76
Sprint−2(20) <s> = 0.03
Zhewan−1(10) <s> = 0.03
Zhewan−1(25) <s> = 0.76
Zhongwan−6(10) <s> = 0.15
Zhongwan−6(25) <s> = 0.04

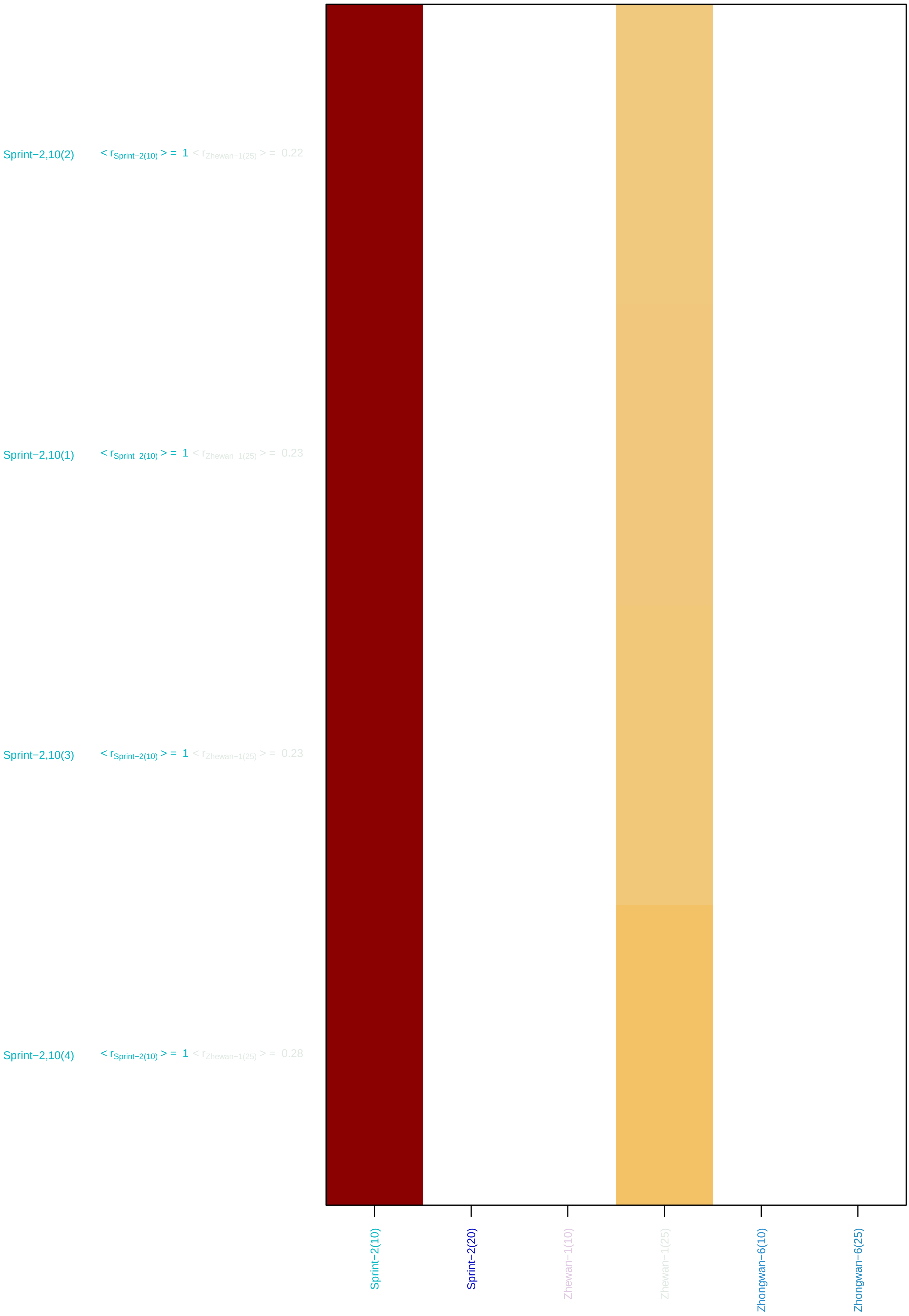
Sprint−2,10(2)
< r_Sprint−2(10) > = 1 < r_Zhewan−1(25) > = 0.22
Sprint−2,10(1)
< r_Sprint−2(10) > = 1 < r_Zhewan−1(25) > = 0.23
Sprint−2,10(3)
< r_Sprint−2(10) > = 1 < r_Zhewan−1(25) > = 0.23
Sprint−2,10(4)
< r_Sprint−2(10) > = 1 < r_Zhewan−1(25) > = 0.28
Sprint−2(10)
Sprint−2(20)
Zhewan−1(10)
Zhewan−1(25)
Zhongwan−6(10)
Zhongwan−6(25)

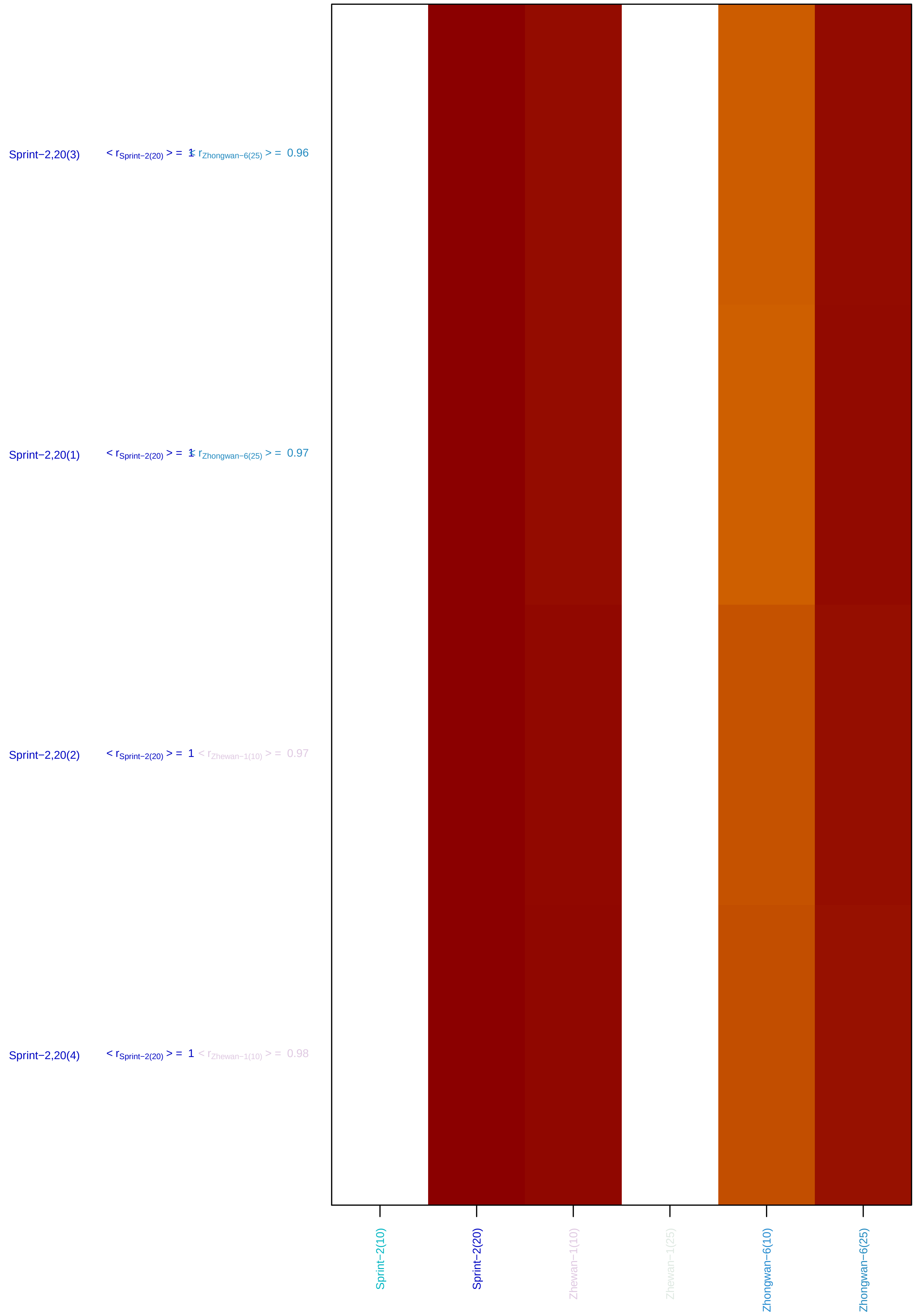

Sprint−2,20(3)
< r Sprint−2(20) > = 1
< r Zhongwan−6(25) > = 0.96
Sprint−2,20(1)
< r Sprint−2(20) > = 1
< r Zhongwan−6(25) > = 0.97
Sprint−2,20(2)
< r Sprint−2(20) > = 1
< r Zhewan−1(10) > = 0.97
Sprint−2,20(4)
< r Sprint−2(20) > = 1
< r Zhewan−1(10) > = 0.98
Sprint−2(10)
Sprint−2(20)
Zhewan−1(10)
Zhewan−1(25)
Zhongwan−6(10)
Zhongwan−6(25)

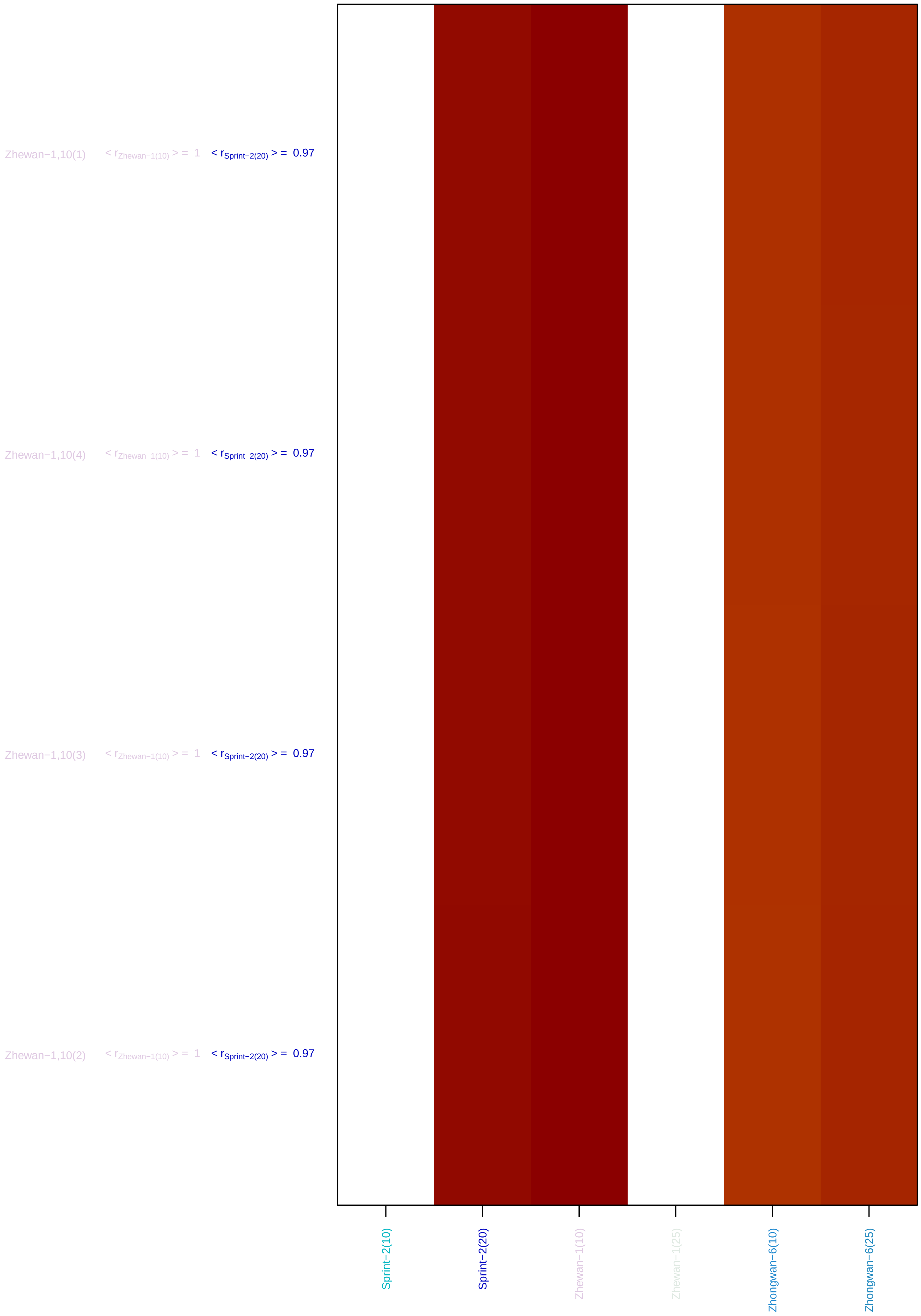

Zhewan−1,10(1)
< r_Zhewan−1(10) > = 1
< r_Sprint−2(20) > = 0.97
Zhewan−1,10(4)
< r_Zhewan−1(10) > = 1
< r_Sprint−2(20) > = 0.97
Zhewan−1,10(3)
< r_Zhewan−1(10) > = 1
< r_Sprint−2(20) > = 0.97
Zhewan−1,10(2)
< r_Zhewan−1(10) > = 1
< r_Sprint−2(20) > = 0.97
Sprint−2(10)
Sprint−2(20)
Zhewan−1(10)
Zhewan−1(25)
Zhongwan−6(10)
Zhongwan−6(25)

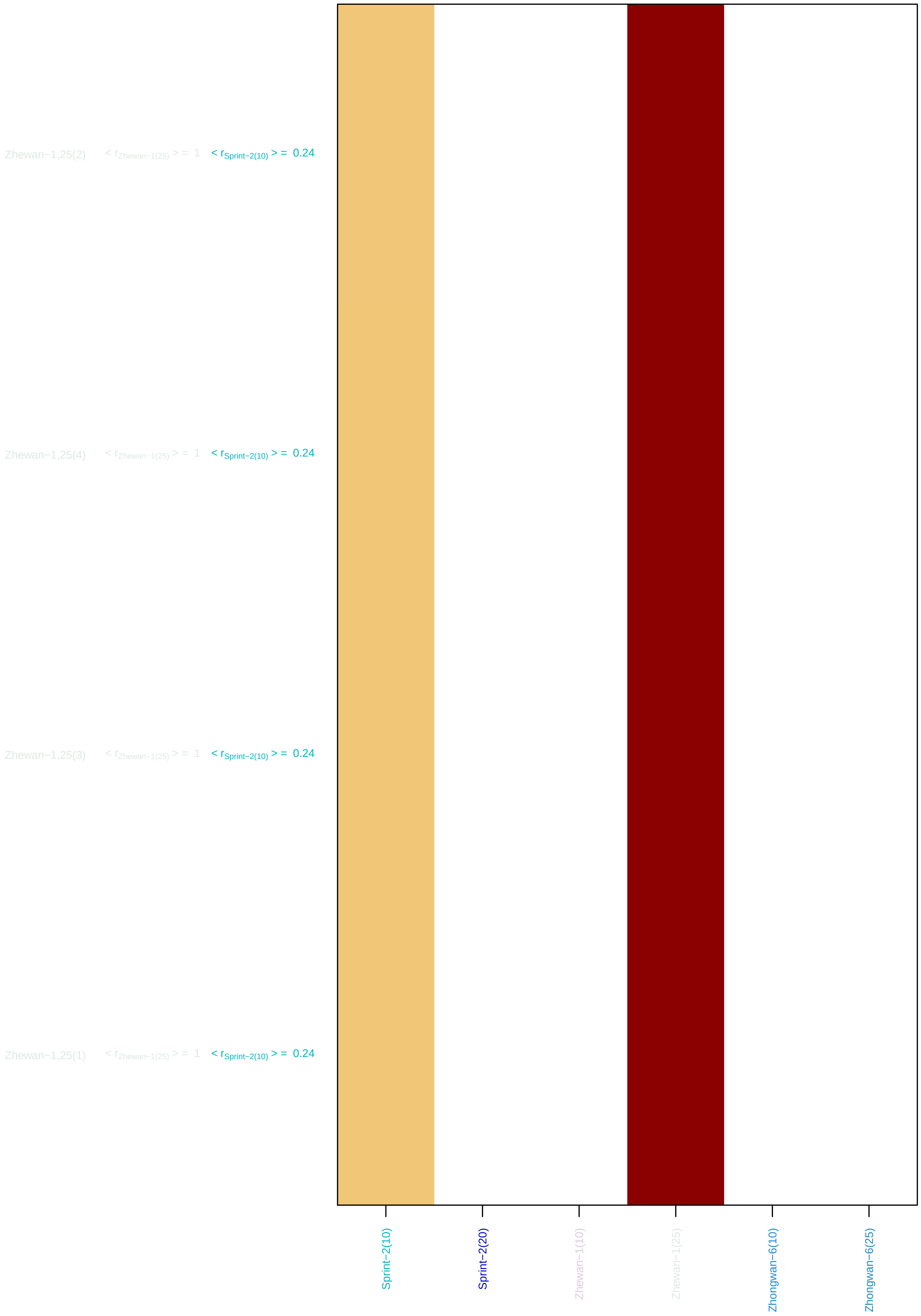

Zhewan−1,25(2)
< r Zhewan−1(25) > = 1
< r Sprint−2(10) > = 0.24
Zhewan−1,25(4)
< r Zhewan−1(25) > = 1
< r Sprint−2(10) > = 0.24
Zhewan−1,25(3)
< r Zhewan−1(25) > = 1
< r Sprint−2(10) > = 0.24
Zhewan−1,25(1)
< r Zhewan−1(25) > = 1
< r Sprint−2(10) > = 0.24
Sprint−2(10)
Sprint−2(20)
Zhewan−1(10)
Zhewan−1(25)
Zhongwan−6(10)
Zhongwan−6(25)

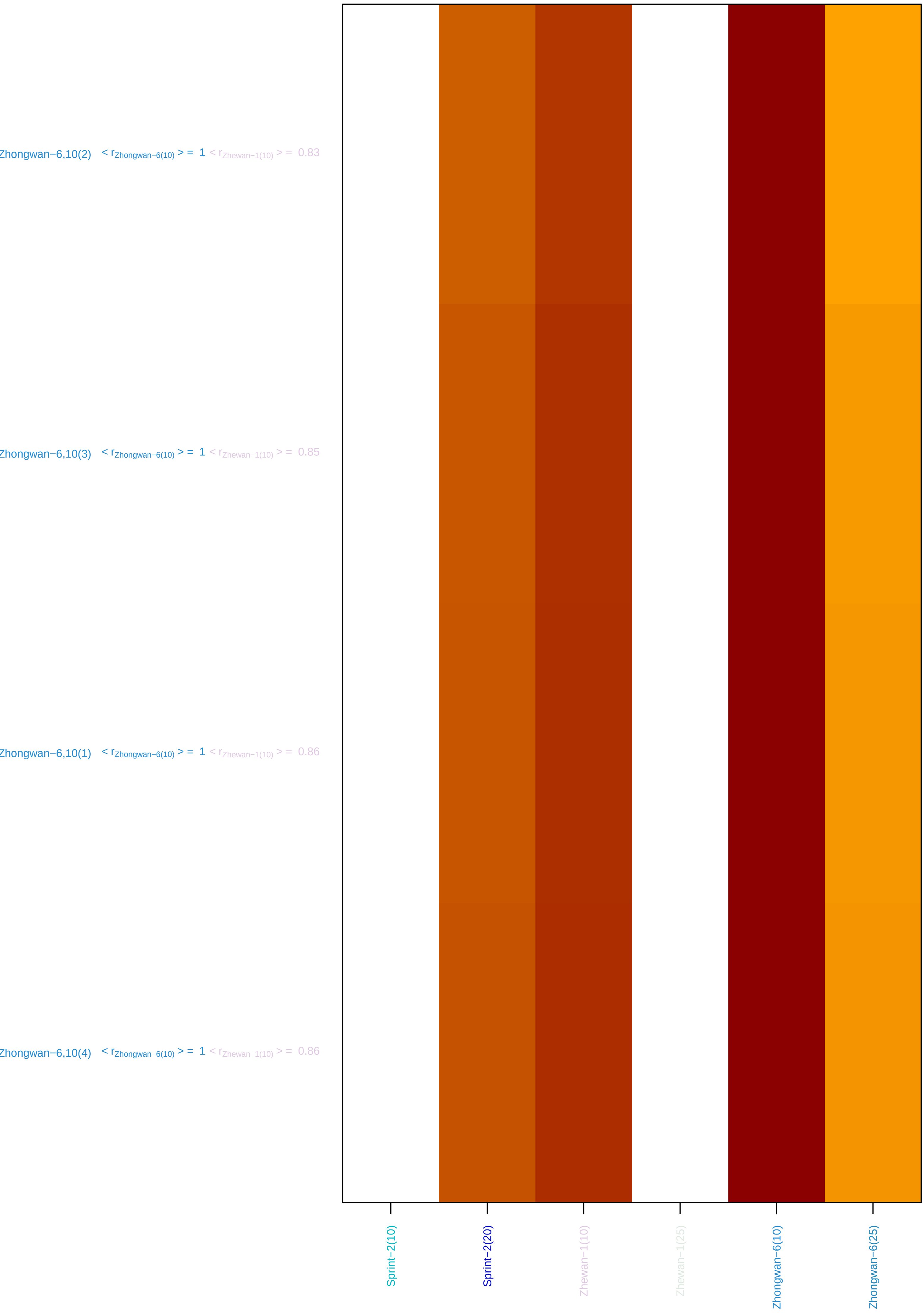

Zhongwan−6,10(2) < $r_{Zhongwan-6(10)}$ > = 1 < $r_{Zhewan-1(10)}$ > = 0.83
Zhongwan−6,10(3) < $r_{Zhongwan-6(10)}$ > = 1 < $r_{Zhewan-1(10)}$ > = 0.85
Zhongwan−6,10(1) < $r_{Zhongwan-6(10)}$ > = 1 < $r_{Zhewan-1(10)}$ > = 0.86
Zhongwan−6,10(4) < $r_{Zhongwan-6(10)}$ > = 1 < $r_{Zhewan-1(10)}$ > = 0.86
Sprint−2(10)
Sprint−2(20)
Zhewan−1(10)
Zhewan−1(25)
Zhongwan−6(10)
Zhongwan−6(25)

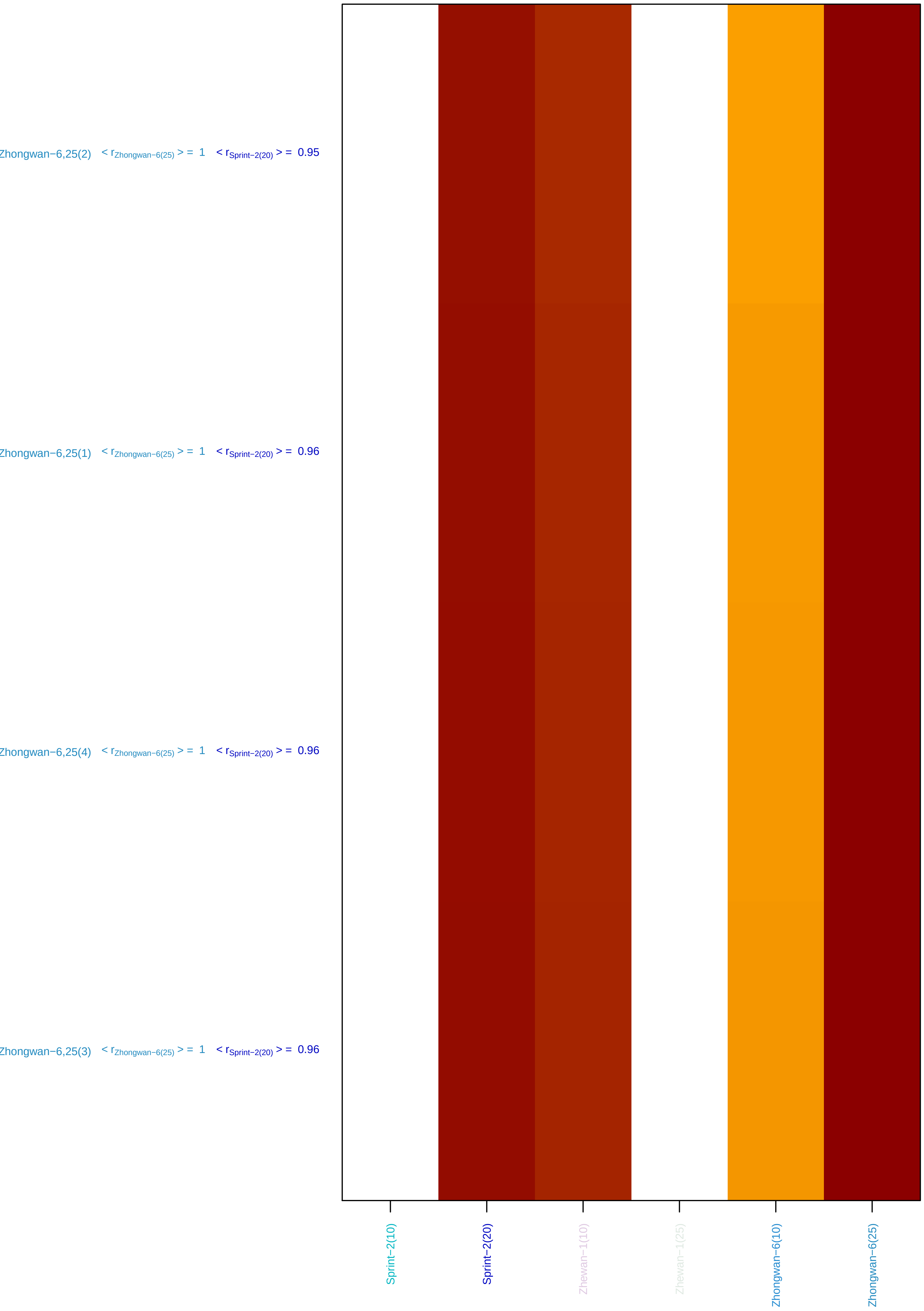

Zhongwan−6,25(2) < r_Zhongwan−6(25) > = 1 < r_Sprint−2(20) > = 0.95
Zhongwan−6,25(1) < r_Zhongwan−6(25) > = 1 < r_Sprint−2(20) > = 0.96
Zhongwan−6,25(4) < r_Zhongwan−6(25) > = 1 < r_Sprint−2(20) > = 0.96
Zhongwan−6,25(3) < r_Zhongwan−6(25) > = 1 < r_Sprint−2(20) > = 0.96
Sprint−2(10)
Sprint−2(20)
Zhewan−1(10)
Zhewan−1(25)
Zhongwan−6(10)
Zhongwan−6(25)
